# Optogenetic manipulation of cardiac repolarization gradients using sub-threshold illumination

**DOI:** 10.3389/fphys.2023.1167524

**Published:** 2023-05-05

**Authors:** Gerard A. Marchal, Valentina Biasci, Leslie M. Loew, Annibale Biggeri, Marina Campione, Leonardo Sacconi

**Affiliations:** ^1^ European Laboratory for Non-Linear Spectroscopy—LENS, Florence, Italy; ^2^ National Institute of Optics (INO-CNR), Florence, Italy; ^3^ Institute of Clinical Physiology (IFC-CNR), Pisa, Italy; ^4^ Department of Experimental and Clinical Medicine, University of Florence, Florence, Italy; ^5^ Center for Cell Analysis and Modeling, University of Connecticut, Farmington, CT, United States; ^6^ Department of Cardiac, Thoracic, Vascular Sciences and Public Health, University of Padua, Padua, Italy; ^7^ Institute of Neuroscience (IN-CNR) and Department of Biomedical Science University of Padua, Padua, Italy; ^8^ Institute for Experimental Cardiovascular Medicine, University Heart Center and Medical Faculty, University of Freiburg, Freiburg, Germany

**Keywords:** optogenetics, sub-threshold illumination, optical mapping, electrophysiological modulation, repolarization gradients

## Abstract

**Introduction:** Mechanisms underlying cardiac arrhythmias are typically driven by abnormalities in cardiac conduction and/or heterogeneities in repolarization time (RT) across the heart. While conduction slowing can be caused by either electrophysiological defects or physical blockade in cardiac tissue, RT heterogeneities are mainly related to action potential (AP) prolongation or abbreviation in specific areas of the heart. Importantly, the size of the area with altered RT and the difference between the short RT and long RT (RT gradient) have been identified as critical determinators of arrhythmogenicity. However, current experimental methods for manipulating RT gradient rely on the use of ion channel inhibitors, which lack spatial and temporal specificity and are commonly only partially reversible. Therefore, the conditions facilitating sustained arrhythmia upon the presence of RT heterogeneities and/or defects in cardiac conduction remain to be elucidated.

**Methods:** We here employ an approach based on optogenetic stimulation in a low-intensity fashion (sub-threshold illumination), to selectively manipulate cardiac electrical activity in defined areas of the heart.

**Results:** As previously described, subthreshold illumination is a robust tool able to prolong action potentials (AP), decrease upstroke velocity as well as slow cardiac conduction, in a fully reversible manner. By applying a patterned sub-threshold illumination in intact mouse hearts constitutively expressing the light-gated ion channel channelrhodopsin-2 (ChR2), we optically manipulate RT gradients and cardiac conduction across the heart in a spatially selective manner. Moreover, in a proof-of-concept assessment we found that in the presence of patterned sub-threshold illumination, mouse hearts were more susceptible to arrhythmias. Hence, this optogenetic-based approach may be able to mimic conduction slowing and RT heterogeneities present in pathophysiological conditions.

## 1 Introduction

Cardiovascular diseases are the most common cause of death worldwide, and a substantial number of these deaths are caused by cardiac arrhythmias resulting in sudden cardiac death (Adabag et al., 2010). Mechanisms underlying cardiac arrhythmias are typically driven by abnormalities in cardiac conduction and/or repolarization. Conduction slowing can be caused by either electrophysiological defects such as reduced sodium current or physical blockade by myocardial fibrosis, and poses a well-established risk for generation of re-entrant arrhythmia (de Jong et al., 2011; Marchal and Remme, 2022). In addition, heterogeneities in repolarization time (RT) have been identified as pro-arrhythmic. RT heterogeneities are caused by action potential (AP) prolongation or abbreviation in specific areas of the heart, which can be caused by inherited disease (i.e., genetic disorders causing ion channel dysfunction), but also occur secondary to acquired heart disease such as myocardial infarction (Kelemen et al., 2022). Previously, the impact of RT heterogeneities on arrhythmia has been established in transgenic mouse models (Jeron et al., 2000; Salama et al., 2009), as well as upon infusion with RT-altering drugs (Fabritz et al., 2003) and/or blockade of the atrio-ventricular node in mouse, rabbit, and dog (Milberg et al., 2004; Loen et al., 2022). However, these approaches lack the ability to control the size of the affected area, often resulting in RT alterations across the whole ventricles. Importantly, the size of the area with altered RT and the difference between the short RT and long RT (RT gradient) have been identified as critical determinators of arrhythmogenicity (Cluitmans et al., 2021; Rivaud et al., 2021). However, current experimental methods to modulate repolarization locally rely on the infusion of AP duration (APD)-modulating drugs in single coronary arteries, limiting versatility of the spatial characteristics of the area with altered APD and restricting studies to larger animal models. In addition, the ion channel inhibitors presently used in most experimental methods for manipulating RT lack spatial and temporal specificity and are commonly only partially reversible. Therefore, aspects of the conditions facilitating sustained arrhythmia upon the presence of RT heterogeneities remain to be elucidated.

Recent optogenetic light-based pacing strategies enabled modulation of cardiac activity in a spatial- and temporal-specific manner, employing mice expressing the light-activated ion channel channelrhodopsin-2 (ChR2) (Entcheva and Kay, 2021). When activated by a light source, this unspecific ion channel allows positively charged ions to enter cardiomyocytes, inducing depolarization of the membrane potential (Nagel et al., 2005). Light-based pacing strategies have been proposed as an alternative for wired electrical stimulation for cardiac pacing (Bruegmann et al., 2010; Nussinovitch and Gepstein, 2015), as well as conventional internal cardioverters (Crocini et al., 2016; Nyns et al., 2017; Bruegmann et al., 2018). Moreover, optogenetics are a robust tool for investigating wave dynamics in cardiac tissue, studying the mechanisms underlying the induction, maintenance and control of cardiac arrhythmias (Burton et al., 2015; Feola et al., 2017). Importantly, optogenetic interventions have so far mostly been used for generating transient and intense depolarizing currents for triggering APs and cardioversion. Conversely, we and others recently described the ability of optogenetics for imposing a continuous depolarizing current with amplitudes that are too low to elicit APs (sub-threshold illumination), to modulate activation and repolarization characteristics in defined areas of the heart (Karathanos et al., 2014; Biasci et al., 2022). As such, this approach may be utilised to mimic conduction slowing and RT heterogeneities present in pathophysiological conditions.

Accordingly, to avoid misinterpretation of experimental readouts when applying sub-threshold illumination, cardiac electrical activity should be stable throughout the entire experiment. Since the mechanical uncoupler blebbistatin affects cardiac electrophysiology (Brack et al., 2013), we here first assessed the time-dependent impact of blebbistatin on cardiac repolarization and conduction characteristics when applied at a conventional concentration and a lower concentration in isolated murine hearts. Subsequently, we investigate the extent of RT gradients, local conduction slowing, and pro-arrhythmia upon application of sub-threshold optogenetic stimulation by using different illumination patterns.

## 2 Materials and methods

### 2.1 Mouse model generation

Transgenic mice (ChR2-mhc6-cre+) with cardiomyocyte-specific expression of ChR2 (H134R variant) were generated as previously described (Zaglia et al., 2015) and employed in this study. All animal handling and procedures were performed in accordance with the guidelines from Directive 2010/63/EU of the European Parliament on the protection of animals used for scientific purposes. The experimental protocol was approved by the Italian Ministry of Health (protocol number 531/2022-PR).

### 2.2 Isolated and perfused mouse hearts

Mice (6 months old) were heparinised (0.1 mL at 5,000 units/mL), anesthetised by isoflurane inhalation (5%), and euthanised by cervical dislocation. The excised heart was immediately bathed in Krebs-Henseleit (KH) solution and cannulated through the aorta. The KH buffer contained (in mM): 120 NaCl, 5 KCl, 2 MgS_2_ O_4_–7H_2_O, 20 NaHCO_3_, 1.2 NaH_2_PO_4_–H_2_O, 1.8 CaCl_2_ and 10 glucose, pH 7.4 when equilibrated with carbogen (95% oxygen and 5% carbon dioxide). Cardiac contraction was inhibited during the entire experiment with 1 or 10 μM (±)-Blebbistatin (Enzo Life Sciences, Farmingdale, NY, United States) in the perfusion and bath solution. The cannulated heart was perfused through the aorta (using a horizontal Langendorff perfusion system) with KH solution and then transferred to a custom-built optical mapping chamber at a constant flow of 2.5 mL/min at 36°C ± 0.5°C. Two platinum electrodes were placed below the heart for monitoring cardiac electrical activity via electrocardiogram (ECG). 1 mL of perfusion solution containing the voltage sensitive dye (VSD) di-4-ANBDQPQ 6 μg/mL, University of Connecticut Health Center, Farmington, CT, United States (Matiukas et al., 2007), was bolus injected into the aorta. All the experiments were performed within 1 h after dye loading to avoid potential re-distribution of the dye and accumulation of phototoxic by-products.

### 2.3 All-optical imaging and manipulation platform

Optical mapping and control were performed using a custom-made mesoscope as previously described (Scardigli et al., 2018). In short, whole mouse hearts were illuminated by a light emitted diode (LED) operating at a wavelength centred at 625 nm (M625L3, Thorlabs, Newton, NJ, United States; maximum intensity of 0.875 mW/mm^2^) in a wide-field configuration using a ×2 objective (TL2x-SAP, Thorlabs, Newton, NJ, United States). A ×20 objective (LD Plan-Neofluar ×20/0.4 M27, Carl Zeiss Microscopy, Oberkochen, Germany) was used to focus the fluorescent signal emitted by the VSD on the central portion (128 × 128 pixels) of the sensor of a sCMOS camera (OrcaFLASH 4.0, Hamamatsu Photonics, Shizuoka, Japan) operating at a frame rate of 1 kHz (1 ms actual exposure time). The detection path allows a field of view (at the object space) of 10.1 × 10.1 mm sampled with a pixel size of 80 µm. Optogenetic illumination was performed by employing a Lightcrafter 4500 projector (Texas Instruments, Dallas, TX, United States), operating at a wavelength of 470 nm, enabling projection of user-defined light patterns onto the cardiac surface. In order to illuminate in a sub-threshold fashion, the threshold for AP initiation was determined for each mouse heart, and illumination was applied a light intensity (LI) below the threshold. Overall, mouse hearts were illuminated with a mean LI of 0.136 ± 0.011 mW/mm^2^. LIs were measured at sample site using a photodiode sensor (PD300-3W, Ophir Optronics, Jerusalem, Israel). User-defined illumination patterns (whole ventricular surface, apex, base, right ventricular area, and left ventricular area of the heart) were applied. Hearts were electrically paced by bipolar electrodes positioned at the apex and at the base attached to an isolated constant voltage stimulator (DS2A, Digitimer, Welwyn Garden City, Hertfordshire, United Kingdom).

### 2.4 Arrhythmia induction

To assess susceptibility to arrhythmia, we employed a stimulation protocol called “parasystole paradigm” where extra beats were electrically induced. Briefly, while leaving the sinus node (SN) intact, mouse hearts were electrically paced at the apex or at the base at a cycle length (CL) of 1.7 × (SN rate). Since each mouse heart has a different SN rate, the pacing CL and protocol duration varied between hearts. During the pacing protocol, cardiac activity was assessed using a two-lead pseudo-electrocardiogram. Arrhythmia was defined as irregular cardiac activity of longer than 250 ms.

### 2.5 Data and image analysis

All programs for data acquisition and analysis were developed with LabVIEW software (National Instruments). For optical recordings, ΔF/F0 imaging of cardiac electrical activity was performed by processing raw data: for each frame, the background was first subtracted, then the voltage-independent bleaching effect during each individual trace was corrected by normalising the fluorescent trace using either a linear or a non-linear polynomial fit applied to the diastolic phase. For each heart, AP kinetics parameters were measured, trace by trace, in order to get the mean values after averaging 5–10 subsequent trials. AP maximum rising slope (APRS), AP repolarization duration (APRD) at 50% of repolarization (APRD_50_), 70% of repolarization (APRD_70_), 90% of repolarization (APRD_90_) were measured in a selected region of interest (ROI) of 10 × 10 pixels (≈1 mm^2^). APRS -which is a measure of the velocity of the AP excitation phase- was normalised for AP amplitude. To dissect the effect of optogenetic stimulation on activation and repolarization kinetics, APRD was determined relative to the time of maximum depolarization. APRS, APRD_50_ and APRD_90_ maps were generated after a spatial binning of 4 × 4 pixels across the whole ventricle. APD_70_ alternans was calculated using the following formula: $(\sum \frac{n-1}{i=1}|{APD}_{70},i+1,-,{APD}_{70},i/\left(n-1\right)$ . Conduction velocity (CV) was calculated after a spatial binning of 6 × 6 pixels using a multi-vector approach: a seed reference pixel was arbitrarily chosen, and the cross-correlation of the fluorescence trace was calculated pixel by pixel, in order to estimate the temporal shift among every pixel (activation map). Next, local velocity maps were generated by calculating the delay between adjacent pixels divided by the pixel size. Since the local direction of the AP wavefront is represented by a vector for each pixel, the mean CV was calculated by averaging local CVs. Wavefront dispersion was determined using the standard deviation of the angle of local AP wavefront propagation vectors generated for each pixel.

In each mouse heart, motion artefact during optical recordings was assessed and reported as displacement of centre of mass (CM) of the heart and variation of heart area. In the first, we estimated the X; Y position of CM in each frame and the corresponding standard deviation (SD) in the stack. Subsequently, we found the displacement of CM in pixels with the following formula $\sqrt{{\left(SDx\right)}^{2}+{\left(SDy\right)}^{2}}$ and we multiplied the result for the pixel size (80 µm). For the variation of heart area, we first calculated the heart area (in mm^2^) in each frame as well as the mean area and the SD in the stack. Afterwards, we normalized the SD to the mean area, and we obtained the variation of heart area in %. Graphical representation of data was obtained using OriginPro 2018, version 9.5 64-bit (OriginLab Corporation, Northampton, MA United States).

### 2.6 Statistics

For each experimental condition, data from each heart was averaged, and this average was used for comparison and statistical analysis. Data is plotted as mean ± standard error of mean (SEM). Plots and statistical analyses were performed in GraphPad Prism software (version 9, GraphPad Software, San Diego, CA, United States) Two-way repeated measures (RM) analysis of variance (ANOVA), and in the case of missing values a Mixed Effects analysis, was used to compare changes in electrophysiological features between the illumination patterns. For the comparison of means at specific CLs, the Tukey’s *post hoc* analysis was used. To investigate the general influence of time on electrophysiological features in 1 μM and 10 µM blebbistatin perfused-mouse hearts, a regression test was additionally applied: an ANOVA test was used to assess if the linear fitting function is significantly better than a constant function. For motion artefact analyses, unpaired Student’s t-tests were used to compare 1 μM and 10 µM blebbistatin perfused mouse hearts.

To analyse arrhythmia susceptibility results, we first assessed the overall effect of sub-threshold illumination on arrhythmia inducibility. To this end, we estimated incidence rates of arrhythmia (total arrhythmia events/protocol duration in seconds) and 95% confidence intervals in the absence and presence of sub-threshold illumination. Subsequently, to evaluate the effect of each illumination pattern on arrhythmia inducibility, we estimated the incidence rate ratio (IRR) and 95% confidence intervals for each illumination pattern, taking absence of sub-threshold illumination as reference—i.e., the IRR was calculated as the incidence rate for each illumination pattern divided by the incidence rate for the control condition. Technically, we performed two comparisons: 1) presence versus absence of illumination, and 2) five illumination patterns versus absence of illumination. The comparisons were all conducted within hearts, since protocol duration differed between hearts. However, the type and number of illumination patterns were not constant among hearts, which created some imbalance and potential confounding. We therefore specified a Poisson mixed effect model (Skrondal and Rabe-Hesketh, 2004; Rabe-Hesketh and Skrondal, 2012), in which a random intercept was applied as an identifier for each heart. The inputs for this model are the frequency of arrythmias, the exposure time to the arrhythmia induction protocol, and the condition, which was the presence/absence of illumination (comparison 1) or the individual illumination patterns vs. control (compassion 2).

The frequency of arrythmias was reported as rate per second and 95% confidence interval based on Poisson likelihood (Clayton and Hills, 2013). Incidence Rate Ratios and 95% confidence intervals was obtained exponentiating the Poisson mixed effects regression coefficient and 95% confidence intervals. Statistical test on regression coefficients were performed by Wald test (Skrondal and Rabe-Hesketh, 2004).

## 3 Results

### 3.1 Blebbistatin at high concentration affects repolarization and conduction kinetics

**FIGURE 1:**
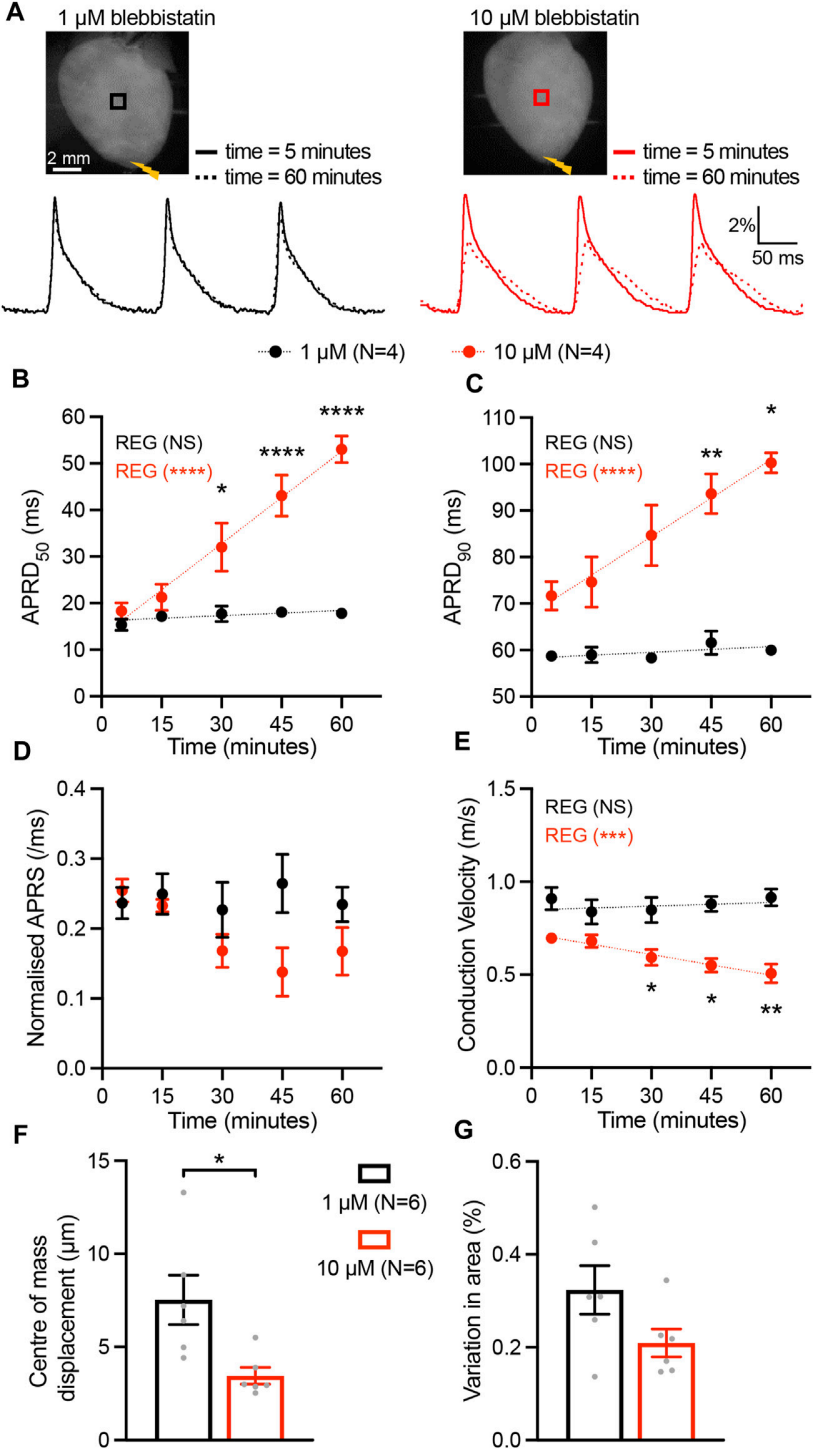
Time-dependent impact of blebbistatin at 1 µM (low) and 10 µM (high) concentrations. **(A)** Top, representative fluorescence images (F0) of mouse hearts perfused with blebbistatin at 1 µM (left) and 10 µM (right) concentrations. Mouse hearts were electrically paced at the apex (yellow bolt symbols) at a cycle length of 150 ms. Bottom, fluorescent signals (ΔF/F) representing action potentials (APs) extracted from the black and red ROIs at time 5 and 60 min after the hearts were stained with the VSD. **(B–E)** Time effect of blebbistatin on AP repolarization duration at 50% and 90% of repolarization (APRD_50_, APRD_90_), AP rising slope (APRS), and conduction velocity (CV). Parameters were measured in the ROIs shown in **(A)**. Data is reported as mean ± SEM and linear fit on experimental data was superimposed. Regression analysis results (REG; ANOVA test) are shown. A Mixed-Effects with RM analysis with Tukey’s post-hoc test was also applied. **(F, G)** The motion artefact of the heart displayed as centre of mass displacement and variation of heart area at different concentrations at blebbistatin. Data is shown as mean ± SEM, N represents number of hearts assessed, and Student’s t-test for mean comparison was applied. *p < 0.05, **p < 0.01, ****p < 0.0001.

To assess the time-dependent impact of the myosin inhibitor blebbistatin on cardiac excitation and repolarization characteristics, we applied blebbistatin at the commonly used (high) concentration of 10 µM as well as at the low concentration of 1 µM in murine hearts. Epicardial optical mapping was performed at 5, 15, 30, 45, and 60 min after VSD loading, during an electrical stimulation at the apex with 150 ms cycle length (CL). Blebbistatin at high concentration resulted in a significant time-dependent prolongation of action potential repolarization duration (APRD) at 50% and 90% of repolarization (APRD_50_, APRD_90_). By contrast, APRD at both repolarization stages remained unaffected when blebbistatin was applied at low dose (Figures 1A–C). Additionally, we found a slight but not significant time-dependent decrease in the AP rising slope (APRS) at high blebbistatin, while unchanged when applying low concentration (Figure 1D). Although only a trend for reduced APRS was observed, the related conduction velocity (CV) was highly significantly reduced over time when applying high concentration blebbistatin, while remaining stable upon low blebbistatin (Figure 1E). Assessment of cardiac movement during recordings revealed that the displacement of the centre of mass was significantly higher when applying low blebbistatin (Figure 1F), while the variation in cardiac surface (i.e., contractile movement) did not significantly differ between the two blebbistatin concentrations (Figure 1G).

### 3.2 Low-intensity illumination leads to reduced AP upstroke velocity and repolarization prolongation

**FIGURE 2:**
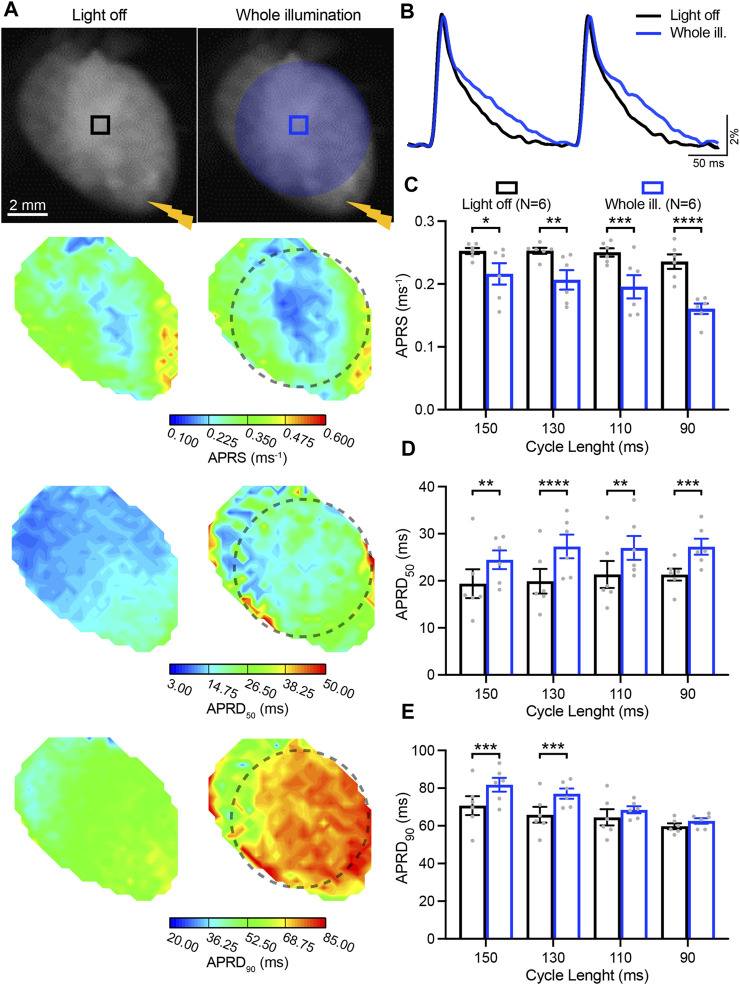
Low-intensity optogenetic stimulation of the entire ventricular surface reduces action potential (AP) upstroke velocity and prolongs AP repolarization duration (APRD). **(A)** Top: representative fluorescence images (F0) of mouse hearts perfused with 1 µM blebbistatin with the illumination pattern indicated. Mouse hearts were electrically paced at the apex (yellow bolt symbol) with a cycle length (CL) of 150, 130, 110, and 90 ms, in the absence (left) and in the presence of sub-threshold illumination of the entire ventricular surface (right). Bottom: representative AP rising slope (APRS) and APRD at 50% and 90% repolarization (APRD_50_, APRD_90_) maps recorded at a pacing CL of 130 ms, with the black dashed line indicating the border of the illumination pattern. **(B)** Representative AP traces recorded at pacing CL of 130 ms. **(C–E)** APRS, APRD_50_ and APRD_90_ as a function of the CL, in the absence and in the presence of sub-threshold illumination. The analysis of these parameters was performed in the ROIs shown in **(A)**. Data is reported as mean ± SEM, N represents number of hearts assessed, a two-way RM measurement ANOVA with Tukey’s post-hoc test was applied. *p < 0.05, **p < 0.01, ***p < 0.001, ****p < 0.0001.

We next investigated the impact of sub-threshold optogenetic stimulation on whole-heart electrophysiological characteristics as a function of the electrically-induced cycle length (CL), by quantifying the AP parameters APRS, APRD_50_, and APRD_90_ in the absence and upon optogenetic stimulation of the entire ventricular surface (Figures 2A, B). Given the time-dependent effect of blebbistatin on cardiac electrical activity, a low concentration of blebbistatin (1 µM) was applied during these experiments. Optogenetic stimulation induced a significant decrease in APRS, especially at faster pacing rates (Figure 2C). In addition, illumination induced a significant prolongation of APRD_50_, but the magnitude of prolongation was not impacted by pacing rate (Figure 2D). Conversely, illumination-induced prolongation of APRD_90_ was highly impacted by pacing rate, displaying decreasing prolongation at higher pacing rates (Figure 2E).

### 3.3 Patterned sub-threshold illumination manipulates ventricular excitation and repolarization gradients

Low-intensity optogenetic stimulation was subsequently applied in various patterns, illuminating the apical, basal, left, and right ventricular surface of hearts. Cardiac activation was initiated by an electrode placed at the apex, which stimulated at intervals of 150, 130, 110, and 90 ms.

**FIGURE 3:**
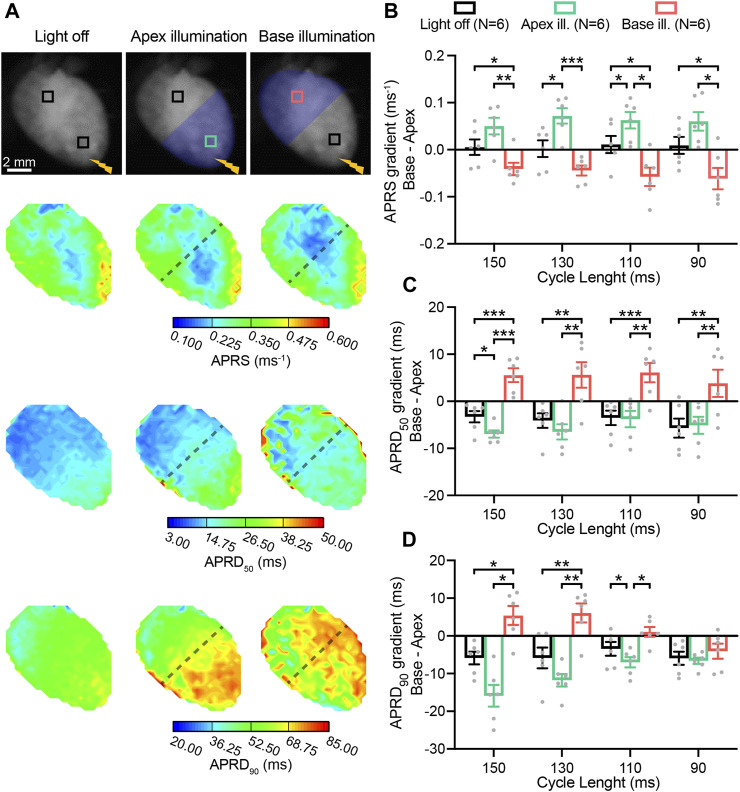
Patterned sub-threshold illumination induces base-to-apex gradients in action potential (AP) activation and repolarization characteristics. **(A)** Top: representative fluorescence images (F0) of mouse hearts perfused with 1 µM blebbistatin, with the illumination pattern indicated. Mouse hearts were electrically paced at the apex (yellow bolt symbol) at a cycle length (CL) of 150, 130, 110, and 90 ms, in the absence and in the presence of patterned sub-threshold illumination (apical or basal ventricular area). Bottom: representative AP rising slope (APRS) and AP repolarization duration at 50% and 90% repolarization (APRD_50_, APRD_90_) maps recorded at a pacing CL of 130 ms, with the black dashed lines indicating the border of the illumination patterns. **(B–D)** Base-to-apex gradient (*base minus apex*) of APRS, APRD_50_ and APRD_90_ as a function of the CL, in the absence and in the presence of patterned sub-threshold illumination. The analysis of these parameters was performed in the ROIs shown in **(A)**. Data is reported as mean ± SEM, N represents number of hearts assessed, a two-way RM measurement ANOVA with Tukey’s post-hoc test was applied. *p < 0.05, **p < 0.01, ***p < 0.001, ****p < 0.0001.

In Figure 3, AP characteristics measured in the basal and apical area, are presented as gradients (*base minus apex*) in absence and presence of patterned sub-threshold illumination at either the apex or base (Figure 3A). APRS was similar between the base and apex when no illumination was applied, while significantly higher in the basal area during apical optogenetic stimulation and *vice versa* (Figure 3B). APRD at different stages of repolarization (APRD_50_, APRD_90_) was slightly longer at the apex when no optogenetic stimulation was applied, which was enhanced upon apical illumination and reversed when the base was illuminated (Figures 3C, D). Specifically, the effects of illumination on APRD_90_ were diminished upon faster pacing. Meanwhile, application of the same illumination patterns while electrically stimulating at the base of the heart led to generally longer APRD at the base, which could be enhanced by basal illumination and diminished by illumination at the apex (Supplementary Figures S1A–C). Similar overall effects were observed when applying blebbistatin at higher concentration, although hearts generally were not able to follow faster pacing rates (Supplementary Figures S2, S3).

**FIGURE 4:**
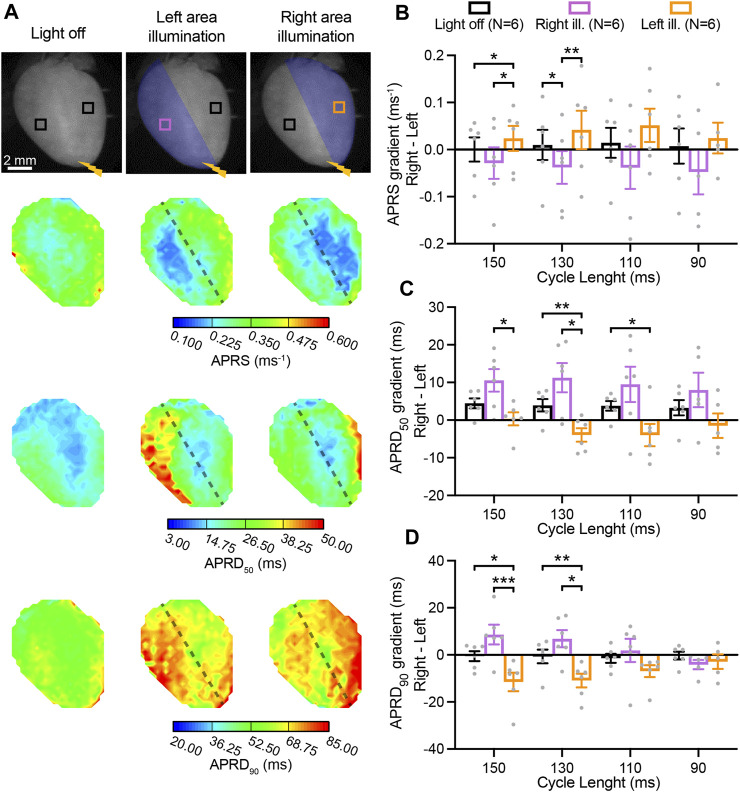
Right-to-left gradients in activation and repolarization action potential (AP) characteristics induced by patterned low-intensity optogenetic stimulation. **(A)** Top: representative fluorescence images (F0) of mouse hearts perfused with 1 µM blebbistatin, indicating the illumination pattern. Mouse hearts were electrically paced at the apex (yellow bolt symbol) with a cycle length (CL) of 150, 130, 110, and 90 ms, in the absence and in the presence of patterned sub-threshold illumination (right- and left- ventricular area). Bottom: representative AP rising slope (APRS) and AP repolarization duration at 50% and 90% repolarization (APRD_50_, APRD_90_) maps recorded at a pacing CL of 130 ms, with black dashed lines indicating the border of the illumination patterns. **(B–D)** Right-to-left gradient (*right minus left*) of APRS, APRD_50_, and APRD_90_ as a function of the CL, in the absence and in the presence of patterned sub-threshold illumination. The analysis of these parameters was performed in the ROIs shown in **(A)**. Data is reported as mean ± SEM, N represents number of hearts assessed, a Mixed-Effects with RM analysis with a Tukey’s post-hoc test was applied. *p < 0.05, **p < 0.01, ***p < 0.001.

We also assessed light-mediated manipulation of gradients between the right- and left-ventricular area (*right minus left*) during illumination of either one of these regions of the heart (Figure 4A). In the absence of illumination, APRS was similar between the right and left. Illumination at the right resulted in a lower APRS at the right as compared to the left, while illumination of the left led to relative decrease in the left (Figure 4B). APRD_50_ was slightly longer at the right, which was enhanced by illumination of the right, while illumination of the left abolished the gradient (Figure 4C). No gradient in APRD_90_ was observed in the absence of illumination, while illumination at the right and left led to the emergence of a repolarization gradient with longer APRD in the illuminated area (Figure 4D). These light-induced gradients were significant at lower pacing rates but diminished at higher pacing rates. Moving electrical stimulation from the apex to the base and applying the same illumination patterns resulted in similar results for APRS, while less capable to induce gradients in APRD (Supplementary Figures S1E–G). Hearts perfused with solution containing high blebbistatin displayed similar right-to-left gradients in AP parameters (Supplementary Figures S2, S3).

In addition to inducing intraventricular gradients in AP activation and repolarization kinetics, patterned sub-threshold led to a trend to pattern-dependent localised alternans (beat-to-beat variations) in AP repolarization duration at 70% repolarization (APRD_70_). These findings are presented in the Supplemental Material (Supplementary Figure S4).

### 3.4 Sub-threshold optogenetic modulation of conduction parameters

**FIGURE 5:**
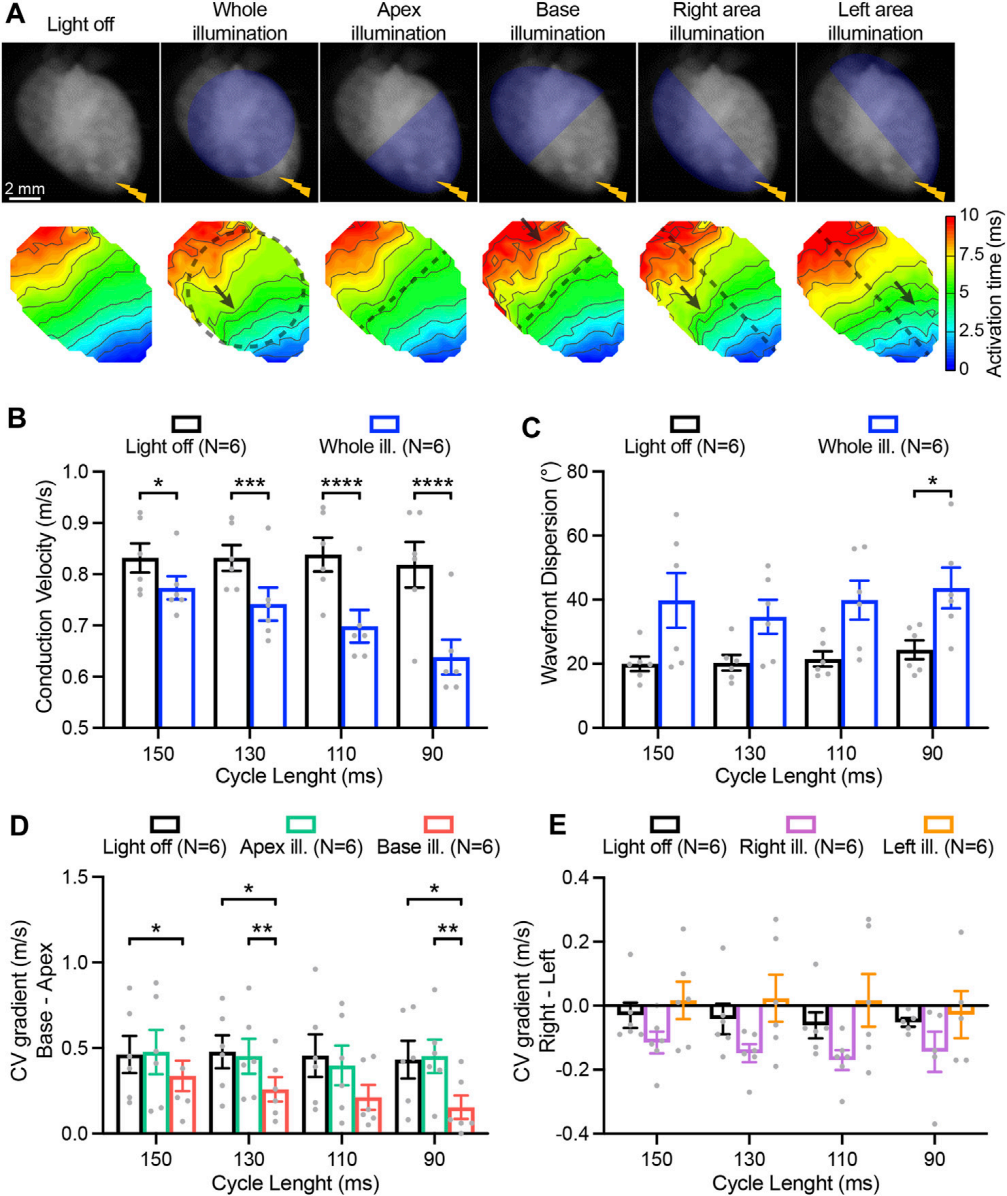
Impact of patterned sub-threshold illumination on ventricular conduction characteristics. **(A)** Top: representative fluorescence images (F0) of mouse hearts perfused with 1 µM blebbistatin showing the illumination protocol. Mouse hearts were electrically paced at the apex (yellow bolt symbol) at a cycle length (CL) of 150, 130, 110, and 90 ms, in the absence and in the presence of patterned sub-threshold illumination. Bottom: representative activation maps recorded at a pacing CL of 130 ms, with black dashed lines indicating the border of the illumination patterns and arrows highlighting wavefront abnormalities. **(B, C)** Conduction velocity (CV) and AP wavefront dispersion as a function of the CL during whole ventricular area illumination. **(D, E)** Base-to-apex and right-to-left gradient of CV as a function of the CL, in the absence and in the presence of patterned sub-threshold illumination. Data is reported as mean ± SEM, N represents number of hearts assessed. A two-way RM measurement ANOVA **(B–D)** or Mixed-Effects with RM analysis **(E)** with Tukey’s post-hoc test was applied. *p < 0.05, **p < 0.01, ***p < 0.001, ****p < 0.0001.

In addition to AP parameters, conduction characteristics were also assessed while performing electrical stimulation at the apex and applying various illumination patterns Figure 5A. Sub-threshold illumination of the entire ventricular surface induced a significant reduction of the CV at every pacing rate (Figure 5B), and was increasingly evident at higher pacing rates. AP wavefront dispersion, which is measure of conduction homogeneity, was overall significantly increased upon illumination, and was especially detectable at the fastest pacing rate (Figure 5C). Hence, sub-threshold illumination affects the speed as well as the directionality of wavefront propagation. Apical illumination had no effect on CV gradients between base and apex (*base minus apex*) which were already present in the absence of illumination, with relatively higher CV at the base. By contrast, illumination at the base did affect the gradient in CV, diminishing the pre-existing gradient (Figure 5D). While illumination at the right- or left-ventricular area led to a general effect on right-to-left (*right minus left*) CV gradients (*p* < 0.05), no significant differences between individual illumination patterns were observed (Figure 5E).

### 3.5 Pro-arrhythmogenic effects of low-intensity illumination

**FIGURE 6:**
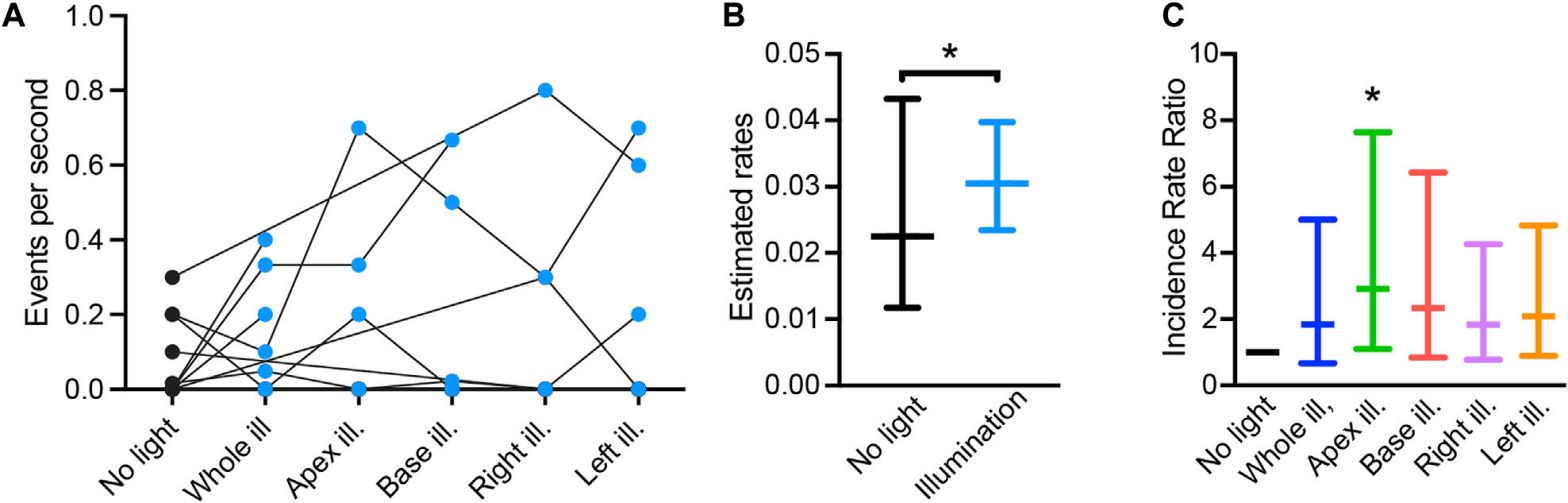
Pro-arrhythmic effect of patterned sub-threshold optogenetic stimulation. **(A)** Arrhythmia events per second recorded during the “parasystole paradigm” stimulation protocol, in the absence (black) and presence (blue) of patterned sub-threshold illumination. **(B)** Overall effect of patterned sub-threshold illumination in arrhythmia inducibility displayed as mean estimated rates of arrhythmia and lower/upper bounds of 95% confidence intervals. **(C)** Effect of individual illumination pattern on arrhythmia inducibility displayed as mean incidence rate ratio (IRR) and lower/upper bounds of 95% confidence intervals. Data was collected from 18 mouse hearts. Wald tests were performed for comparison, *p < 0.05.

Finally, we performed a proof-of-concept assessment on the impact of patterned sub-threshold illumination on arrhythmia inducibility. Arrhythmia susceptibility was assessed by applying a parasystole pacing strategy, while imposing all the previously described illumination patterns (whole ventricular surface, apex, base, right ventricular area, and left ventricular area of the heart), and in the absence of optogenetic stimulation. Arrhythmic events per second in each heart are presented in Figure 6A. Overall, 95% ± 2.7% of the arrhythmias observed were monomorphic, indicating ventricular tachycardia originating from a single rotor. Arrhythmia type was independent from the illumination pattern or stimulation site. Arrhythmia rate was increased when low-intensity illumination was applied, as compared to in the absence of illumination (Figure 6B). When taking individual illumination patterns into account, illumination at the apex resulted in a significant increase in arrhythmia rate (Figure 6C). Hence, patterned sub-threshold illumination results in increased susceptibility to arrhythmia.

## 4 Discussion

### 4.1 Blebbistatin time effect

In epicardial optical mapping experiments, the myosin inhibitor blebbistatin is commonly applied at a concentration of 10 µM as an electromechanical uncoupler to prevent motion artefacts and enhance the quality of the fluorescence traces obtained. We here demonstrate that blebbistatin alters cardiac electrophysiology in a time-dependent manner when applied the commonly used concentration of 10 µM and did not at the lower concentration of 1 µM. Strikingly, previous research highlighted that blebbistatin can precipitate, blocking myocardial microvasculature and thereby inducing ischaemia and metabolic changes (Swift et al., 2012). In addition, a prolonging effect of blebbistatin on repolarization has been described in isolated hearts from rabbit (Brack et al., 2013) and pig (Lee et al., 2019), although other studies report no impact of blebbistatin on AP parameters (Fedorov et al., 2007; Marchant, et al., 2022). We here provide evidence of a time-dependent impact of high-dose blebbistatin on activation and repolarization kinetics in intact mouse hearts. While we did find a significant increase in displacement of centre of mass of the heart when applying blebbistatin at the low concentration, this displacement of ∼4 µm can be considered negligible considering that the pixel size is 80 µm and movement is therefore minimal. Accordingly, we used a low concentration of blebbistatin during the patterned sub-threshold illumination experiments, since cardiac electrical activity should be stable and constant during the entire recording time to avoid misinterpretations of experimental results.

Importantly, even minor residual movement may severely impact signal quality in the setting of uneven staining or tissue heterogeneities (Zasadny et al., 2020). To counter this issue, motion-stabilising algorithms can be applied to correct for movement (Christoph and Luther, 2018). Alternatively, limitations of blebbistatin can potentially also be avoided by using a photostable nitro-derivate of blebbistatin which was recently synthesised and characterised both *in vitro* and *in vivo* (Képiró et al., 2014). Additionally, para-amino-blebbistatin was recently described, and has already been successfully applied to inhibit contraction of zebrafish hearts (Várkuti et al., 2016; van Opbergen et al., 2018). As these novel compounds showed neither phototoxic nor cytotoxic effects, they are promising replacements of blebbistatin for myosin inhibition.

### 4.2 Sub-threshold induced repolarization gradients: relevance for arrhythmia

For decades, it has been known that ventricular heterogeneities in conduction and RT predisposes to arrhythmia, and is specifically associated with idiopathic VF (Peeters et al., 1998). In addition, repolarization heterogeneities secondary to healed myocardial infarction have been identified as causal factors for ventricular tachycardia in porcine and human hearts (Callans and Donahue, 2022; Kelemen et al., 2022). Recent studies further elucidated the requirements to set the stage for re-entry arrhythmia secondary to repolarization heterogeneities, demonstrating the crucial balance between the ventricular area with short RT and long RT, as well as the steepness of the gradient in RT between these two areas (Cluitmans et al., 2021; Rivaud et al., 2021).

Utilising an optogenetic approach and applying sub-threshold illumination, we here identified several illumination patterns which were able to induce distinct gradients in APRD_90_, which would also cause a gradient in effective refractory period (Knollmann et al., 2001). The largest gradient in APRD_90_ was induced by illuminating the apex and resulted in a gradient of ∼15 ms in APRD_90_ between the apical and basal regions of the heart. Previous studies in a porcine model, in which RT gradients were induced by the infusion of drugs specific coronary arteries reported significantly larger RT gradients, with average gradients achieved reaching around 80 ms (Rivaud et al., 2021; van der Waal et al., 2022). However, total repolarization is also considerably longer in porcine hearts as compared to murine hearts. Remarkably, the relative gradient in RT achieved in porcine hearts was similar to the relative gradient in APRD_90_ we achieved by sub-threshold illumination, with a difference of around 25% between the area with long RT and short RT. As such, while the absolute difference in RT achieved by sub-threshold optogenetic stimulation seems limited, it is still possible that the induced RT gradient is sufficient to increase arrhythmia susceptibility and maintenance. Indeed, we observed an increase in arrhythmia susceptibility when sub-threshold illumination patterns were applied. Strikingly, the highest propensity to arrhythmias was observed upon illumination of the apex, the pattern which also resulted in the largest gradient in APRD_90_. Hence, the findings of the present study indicate that the RT gradients induced by sub-threshold optogenetic stimulation is sufficient to enhance arrhythmogenicity.

### 4.3 Limitations and future directions

While we here demonstrate that patterned sub-threshold illumination enables optical manipulation of repolarization gradients and cardiac conduction, the generation of steep gradients proved challenging. An essential contributor to this limitation is the scattering of the stimulation light, resulting in contamination in the border area, as well as a higher degree of stimulation at the epicardial surface than at the endocardium, where the intensity drops with approximately 30% (Crocini et al., 2016). To overcome the limitations imposed by the scattering of blue light in the myocardial tissue, a red-shifted opsin could be adopted for optogenetic manipulation of transmural gradient. Another limiting factor is the non-uniform illumination due to the shape of the heart: although the projector produces light relatively homogeneously, light intensity at the epicardial surface will be heterogeneous due to the curvature of the heart.

Importantly, a recent study performed in pig hearts, demonstrate that a sharp border between the short and long repolarization regions is fundamental for arrhythmia maintenance (Rivaud et al., 2021). While the small dimension of murine hearts was potentially a limiting factor for the generation of gradients, we found an increase in arrhythmia susceptibility when the patterned sub-threshold illumination was applied. However, the driving mechanism and nature of the arrhythmias observed in the current investigation remain elusive. Therefore, a more in-depth analysis on the behaviour of these arrhythmia is required to further elucidate the impact of patterned sub-threshold illumination on arrhythmia maintenance.

We here propose the implementation of patterned sub-threshold stimulation for the modelling of cardiac arrhythmogenic syndromes. However, it is important to note the multifactorial nature of cardiac pathophysiology, which generally not only affects electrophysiological properties of the heart, but also structural, metabolic, and transcriptional aspects. Hence, while sub-threshold optogenetic stimulation models the RT prolongation observed in heart failure, other important pathological factors are lacking (Coronel et al., 2013). Moreover, while the introduced approach can be highly relevant for the investigation of RT gradients secondary to an ischaemic event -which leads to local shortening of RT-(Kelemen et al., 2022), the impact of local sub-threshold illumination is the reverse. Taken together, our findings petition for future investigations focussing on testing optogenetic manipulation of conduction and RT gradients in murine, as well as larger hearts and in more complex geometries. In this respect, panoramic (Qu et al., 2007) or volumetric (Sacconi et al., 2022) imaging could provide more comprehensive knowledge of RT gradient-based mechanisms underlying arrhythmia inducibility and maintenance. These techniques allow for investigation across the whole heart surface as well as within ventricle walls, expanding the epicardial observations reported in this study.

## 5 Conclusion

We here present a novel all-optical method as a robust tool to manipulate cardiac conduction and repolarization characteristics in a spatially-specific manner. An initial assessment indicated that this optogenetic-based strategy resulted in an increase of arrhythmia susceptibility. Hence, this approach represents a promising tool to create a pro-arrhythmogenic substrate driven by conduction slowing and RT heterogeneities. In addition, we provide evidence that the myosin inhibitor blebbistatin alters cardiac electrophysiological characteristics in a time-dependent manner, underlining that blebbistatin should be used in an as low as possible concentration.

## Data Availability

The original contributions presented in the study are included in the article/Supplementary Material, further inquiries can be directed to the corresponding author.
