# Supplementary material for: Optogenetic manipulation of cardiac repolarization gradients using sub-threshold illumination
Source: Front Physiol. 2023 May 5;14:1167524. doi: 10.3389/fphys.2023.1167524 (PMC10196067; doi:10.3389/fphys.2023.1167524)
Supplement: Supplementary file 1 [file DataSheet1.docx]

Supplementary Material

Optogenetic manipulation of cardiac repolarization gradients using sub-threshold illumination

Gerard A. Marchal^1,2,†^, Valentina Biasci^1,3,†^, Leslie M. Loew^4^, Annibale Biggeri^5^, Marina Campione^6^, Leonardo Sacconi^7,8*^

**^†^ These authors contributed equally to this work and share first authorship**

*** Correspondence:** Leonardo Sacconi: leonardo.sacconi@cnr.it

^1^ European Laboratory for Non-Linear Spectroscopy - LENS, Sesto Fiorentino, Florence, Italy

^2^ National Institute of Optics (INO-CNR), Sesto Fiorentino, Florence, Italy

^3^ Department of Experimental and Clinical Medicine, University of Florence, Florence, Italy

^4^ Center for Cell Analysis and Modeling, University of Connecticut, Farmington, CT, USA

^5^ Department of Cardiac, Thoracic, Vascular Sciences and Public Health, University of Padua, Padua, Italy

^6^ Institute of Neuroscience (IN-CNR) and Department of Biomedical Science University of Padua; Padua, Italy

^7^ Institute of Clinical Physiology (IFC-CNR), Pisa, Italy

^8^ Institute for Experimental Cardiovascular Medicine, University Heart Center and Medical Faculty, University of Freiburg, Freiburg, Germany

# Supplementary Figures

**Supplemental Figure S1:** Gradients in mouse hearts perfused with 1 µM blebbistatin, with electrical stimulation at the base, paced at cycle lengths of 150, 130, 110 and 90 ms, in the absence and presence of patterned sub-threshold illumination A-C) Base-to-apex gradients (base minus apex) of action potential (AP) rising slope (APRS), AP repolarization duration at 50% and 90% of repolarization (APRD_50_, APRD_90_) as a function of the CL, in the absence and in the presence of patterned sub-threshold illumination (apical- and basal- ventricular area). D-F) Right-to-left gradient (right minus left) of APRS, APRD_50_ and APRD_90_ as a function of the CL, in the absence and in the presence of patterned sub-threshold illumination (right- and left- ventricular area). Data is reported as mean ± SEM and a Mixed Effects analysis with Tukey’s post-hoc test was applied. **P* < 0.05, ***P* < 0.01, ****P* < 0.001, *****P* < 0.0001.

**Supplemental Figure S2:** Gradients in mouse hearts perfused with 10 µM blebbistatin, with electrical stimulation at the apex. Pacing was done at cycle lengths of 150, 130, and 110 ms, in the absence and in the presence of patterned sub-threshold illumination A-C) Base-to-apex gradient (base minus apex) of action potential (AP) rising slope (APRS), AP repolarization duration at 50% and 90% of repolarization (APRD_50_, APRD_90_) as a function of the CL, in the absence and in the presence of patterned sub-threshold illumination (apical- and basal- ventricular area). D-F) Right-to-left gradient (right minus left) of APRS, APRD_50_ and APRD_90_ as a function of the CL, in the absence and in the presence of patterned sub-threshold illumination (right- and left- ventricular area). Data is reported as mean ± SEM and a Mixed Effects analysis with Tukey’s post-hoc test was applied. **P* < 0.05, ***P* < 0.01, ****P* < 0.001, *****P* < 0.0001.

**Supplemental Figure S3:** Gradients in mouse hearts perfused with 10 µM blebbistatin, with electrical stimulation at the base. Pacing was done at cycle lengths of 150, 130, and 110 ms, in the absence and in the presence of patterned sub-threshold illumination A-C) Base-to-apex gradient (base minus apex) of action potential (AP) rising slope (APRS), AP repolarization duration at 50% and 90% of repolarization (APRD_50_, APRD_90_) as a function of the CL, in the absence and in the presence of patterned sub-threshold illumination (apical- and basal- ventricular area). D-F) Right-to-left gradient (right minus left) of APRS, APRD_50_ and APRD_90_ as a function of the CL, in the absence and in the presence of patterned sub-threshold illumination (right- and left- ventricular area). Data is reported as mean ± SEM and a Mixed Effects analysis with Tukey’s post-hoc test was applied. **P* < 0.05, ***P* < 0.01, ****P* < 0.001, *****P* < 0.0001.

**Supplemental Figure S4:** Localised alternans (beat-to-beat variation) in action potential duration at 70% repolarization (APRD_70_) induced by patterned sub-threshold illumination. Hearts were perfused with 1 µM blebbistatin and pacing was done at cycle lengths (CL) of 150, 130, 110, and 90 ms, in the absence and in the presence of patterned sub-threshold illumination A) Base-to-apex gradient (base minus apex) of APRD_70_ alternans as a function of the CL, in the absence and in the presence of patterned sub-threshold illumination (apical- and basal- ventricular area). B) Absolute APRD_70_ alternans at the basal and apical region at a pacing CL of 90 ms. C) Right-to-left gradient (right minus left) of APRD_70_ alternans as a function of the CL, in the absence and in the presence of patterned sub-threshold illumination (right- and left- ventricular area). B) Absolute APRD_70_ alternans at the right and left region at a pacing CL of 90 ms. Data is reported as mean ± SEM and a Mixed Effects analysis with Tukey’s post-hoc test was applied. **P* < 0.05.
